# Prognostic nomogram based on pre-treatment HALP score for patients with advanced non-small cell lung cancer

**DOI:** 10.1016/j.clinsp.2024.100371

**Published:** 2024-05-11

**Authors:** Shan Gao, Qin Huang, Suosu Wei, Yanru Lv, Yanyan Xie, Yanrong Hao

**Affiliations:** a Medical Oncology Division 1, Clinical Oncology Center, People' s Hospital of Guangxi Zhuang Autonomous Region, Nanning, Guangxi, China; b Department of Oncology and Chemotherapy, Yulin Red Cross Hospital, Yulin, Guangxi, China; c Department of Scientific Cooperation of Guangxi Academy of Medical Sciences, People's Hospital of Guangxi Zhuang Autonomous Region, Nanning, Guangxi, China; d The First Clinical Medical College, Jinan University, Guangzhou, Guangdong, China

**Keywords:** Non-small cell lung cancer (NSCLC), Hemoglobin-Albumin-Lymphocyte-Platelet Score, Survival analysis, Nomogram, Prediction model

## Abstract

**Background:**

To explore the correlation of pre-treatment Hemoglobin-Albumin-Lymphocyte-Platelet (HALP) score with the prognosis of patients with advanced Non-Small Cell Lung Cancer (NSCLC) undergoing first-line conventional platinum-based chemotherapy.

**Methods:**

In this retrospective cohort study, 203 patients with advanced NSCLC were recruited from January 2017 to December 2021. The cut-off value for the HALP score was determined by Receiver Operating Characteristic (ROC) curve analysis. The baseline characteristics and blood parameters were recorded, and the Log-rank test and Kaplan-Meier curves were applied for the survival analysis. In the univariate and multivariate analyses, the Cox regression analysis was carried out. The predictive accuracy and discriminative ability of the nomogram were determined by the Concordance index (C-index) and calibration curve and compared with a single HALP score by ROC curve analysis.

**Results:**

The optimal cut-off value for the HALP score was 28.02. The lower HALP score was closely associated with poorer Progression-Free Survival (PFS) and Overall Survival (OS). The male gender and other pathological types were associated with shorter OS. Disease progression and low HALP were correlated with shorter OS and PFS. In addition, nomograms were established based on HALP scores, gender, pathology type and efficacy rating, and used to predict OS. The C-index for OS prediction was 0.7036 (95% CI 0.643 to 0.7643), which was significantly higher than the C-index of HALP at 6-, 12-, and 24-months.

**Conclusion:**

The HALP score is associated with the prognosis of advanced NSCLC patients receiving conventional platinum-based chemotherapy, and the nomogram established based on the HALP score has a better predictive capability for OS.

## Highlights

• The authors created a new predictive model based on HALP scores in progressive NSCLC.
• HALP score may be an accurate prognostic factor for PFS and OS in advanced NSCLC.

## Introduction

Lung cancer has been the leading cause of cancer-related death worldwide, especially in the population over 50 years.^1^ According to the National Cancer Center statistics, approximately 238,000 new cases of lung cancer are diagnosed in 2023, and the number of deaths caused by lung cancer is over 120,000.^2^ Non-Small Cell Lung Cancer (NSCLC) accounts for about 85% of lung cancer cases.^3^ It is estimated that greater than 60% of patients with NSCLC have locally advanced or metastatic cancers.^4^ Despite significant advancements in the therapy, early diagnosis and management of NSCLC in recent years, the overall prognosis for advanced NSCLC patients is still unsatisfactory, with the five-year Overall Survival (OS) rate fluctuating between 10%‒15%.^5^ Currently, first-line chemotherapy remains the treatment of choice for these patients.^6^ It is important to better predict the prognosis and survival of these patients in the limited time available for treatment.

Several studies have shown that the Tumor-Node-Metastasis (TNM) staging,^7^ tumor markers,^8^ and clinicopathological type^9^ are associated with the survival and prognosis of advanced NSCLC patients, Nevertheless, a single tumor marker usually fails to reflect the systemic condition. TNM staging and clinicopathological types require complex examinations or pathological biopsies. Therefore, it is crucial to identify a simpler, more reliable, and less costly biomarker that can provide a comprehensive response to the systemic situation to predict the outcome of advanced NSCLC patients undergoing traditional first-line therapy.

The establishment of relevant prediction models is crucial for prognosis assessment, and reliable prediction models can provide important references for the development of individualized treatments for cancer patients and for clinical decision-making. In recent years, the nomogram model has been a hot topic in the biomedical field and rapidly popularized in the clinical research of tumor-related diseases due to its accuracy in predicting the outcome of certain diseases and its simplicity in clinical use.^10^^,^^11^

Hemoglobin-Albumin-Lymphocyte-Platelet (HALP) score is a relatively new index that is calculated based on easily obtainable laboratory parameters including albumin, platelets, lymphocytes, and hemoglobin. This score may reflect the nutrition status and inflammatory status of patients, and there is evidence showing that a higher HALP score predicts a better prognosis of patients with different cancers, including liver cancer, esophageal carcinoma, and colorectal cancer.^12^, ^13^, ^14^

Available studies focused on the relevance of HALP score and NSCLC are mainly limited to patients with early-stage cancers.^15^^,^^16^ The correlation between HALP score and prognosis of patients with advanced NSCLC is still unclear. This study aimed to investigate if the HALP score was correlated with Progression Free Survival (PFS) and OS in patients with advanced NSCLC undergoing first-line chemotherapy. Moreover, a nomogram based on the HALP score was established and validated to provide a more reliable, accurate predictor of patients’ survival.

## Methods

### Study design and population

**Figure 1:**
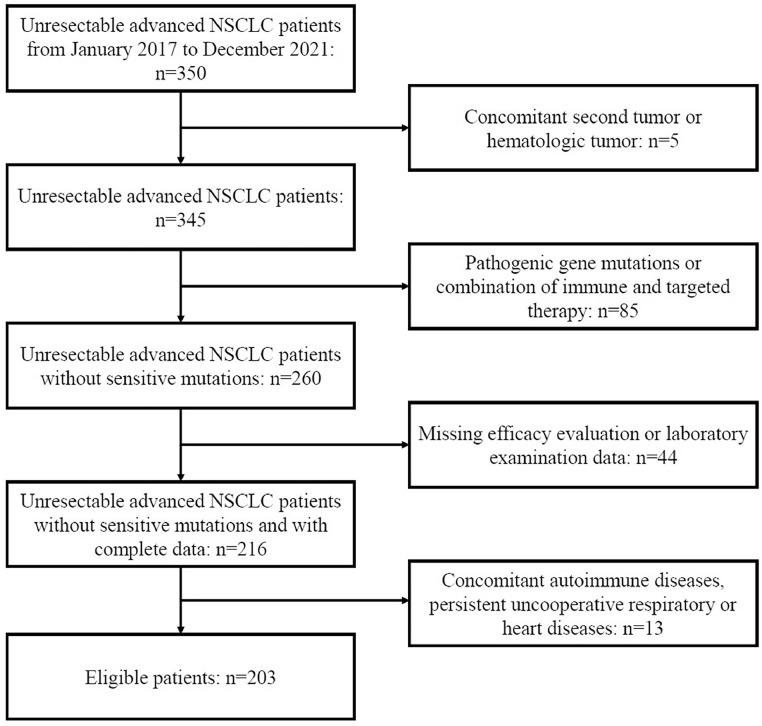
Flow diagram of patient inclusion. NSCLC, Non-Small Cell lung Cancer.

A total of 350 patients with unresectable advanced NSCLC at the People's Hospital of Guangxi Zhuang Autonomous Region between January 2017 and December 2021 were retrospectively reviewed. The inclusion criteria were as follows: 1) Patients had pathologically confirmed NSCLC, 2) Patients had unresectable stage IIIB-IIIC or IV lung cancer with at least 1 measurable lesion (AJCC 8^th^ edition), 3) Patients received platinum-based standard first-line chemotherapy. The exclusion criteria were as follows: 1) Patients had concomitant other tumors; 2) Patients had concomitant autoimmune diseases, persistent uncooperative respiratory or heart diseases; 3) Driver gene mutations were identified, or patients received immune and targeted therapy; 4) Data about the efficacy or laboratory test were missing. Finally, 203 patients were enrolled in this study. The flow diagram is shown in Figure 1.

### Data collection and assessments

The following clinical characteristics were recorded: gender, age, pathological type, stage of diagnosis, pleural effusion status, smoking, regimens for chemotherapy, and Body Mass Index (BMI). Laboratory examinations were conducted 7 days before the first course of chemotherapy, and laboratory findings included Lymphocyte (LYM) count, Hemoglobin (HB) level, Platelet (PLT) count, and Albumin (ALB) level. Age > 65 years was defined as elderly. Patients were classified as underweight (BMI < 18.5 kg/m^2^), normal weight (BMI 18.5‒23.9 kg/m^2^), and overweight (BMI > 24 kg/m^2^). The HALP score was calculated as follows: HB count (g/L) × ALB count (g/L) × LYM count (/L)/PLT count (/L).^17^

According to the criteria for solid tumor efficacy evaluation RECIST 1.1, effectiveness was categorized as Disease Progression (PD), Stable Disease (SD), Partial Remission (PR), or Complete Remission (CR).^18^

Survival status was assessed according to medical records and follow-up by telephone before June 2022. The OS was calculated as the time from the initial diagnosis to the death or the last follow-up. PFS was determined as the time from the date of diagnosis to the first identification of disease progression or death.

### Statistical analysis

The ideal cut-off of HALP score was analyzed by the Receiver Operating Characteristic (ROC) curve analysis. Continuous data are displayed as mean ± Standard Deviation (SD), or median (interquartile range), whereas categorical data as percentage (%). For the baseline characteristics, categorical data were analyzed with the Fisher exact test or Chi-Square test, while continuous variables were with *t*-test or one-way analysis of variance (ANOVA).

Both univariate and multivariate Cox regression analyses were carried out. For multivariate analysis, variables were used as adjustments if they would change the matched Hazard Ratio (HR) by no less than 10% or if they had been significantly linked with OS in the univariate analysis (p < 0.05) or based on the previous findings. Finally, gender, pathological type, smoking status, stage on diagnosis, pleural effusion status, regimens for chemotherapy, therapeutic response, and HALP score were included. Kaplan-Meier curves were used for the survival analysis, and log-rank tests were used for statistical comparisons.

The prognostic nomogram was established according to the multivariate analyses. The calibration curve analysis was performed to evaluate the predictive performance. Comparisons between the new nomogram and HALP score were performed with the software and the performance was evaluated by the C-index and ROC curve. The larger the C-index and Area Under the ROC Curve (AUC), the more accurate the prediction is.^19^

Statistical analysis was performed with the Statistical Software Packages R 3.3.2 (http://www.R-project.org, The R Foundation) and Free Statistics software versions 1.7.^20^ A value of p < 0.05 was considered statistically significant.

## Results

### Cut-off value of HALP score

**Figure 2:**
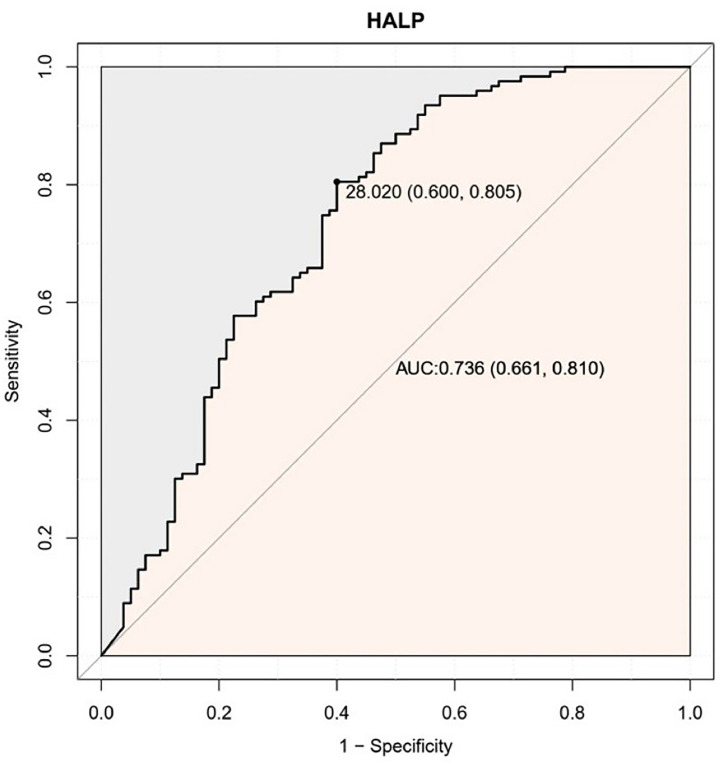
Analysis of HALP score cut-off value with ROC curves. ROC, Receiver Operating Characteristic; HALP, Hemoglobin-Albumin-Lymphocyte-Platelet; AUC, The Area Under the ROC Curve.

ROC curve showed that the ideal cut-off value for HALP score was 28.02 (AUC = 0.736; 95% CI 0.661‒0.810, p < 0.001) (Fig. 2). According to this cut-off value, patients were grouped into HALP-High and HALP-Low groups.

### Clinical characteristics of enrolled patients

Of 350 patients with advanced NSCLC, 203 patients were included for final analysis according to the inclusion and exclusion criteria (Fig. 1). Table 1 displays the initial characteristics by HALP score. The average age was patients were 59.6 ± 9.7 years (range: 30 to 79 years); 137 (67.5%) patients were ≤ 65 years and 66 (32.5%) > 65 years. There were 63 (31.0%) females and 140 (69.0%) males. Pleural effusion was found in 124 (61.1%) patients, and 92 (45.3%) patients had a history of smoking. All patients received platinum-based first-line chemotherapy: pemetrexed + platinum (n = 91, 44.8%), gemcitabine + platinum (n = 15, 7.4%), etoposide + platinum (n = 47, 23.2%), and paclitaxel + platinum (n = 50, 24.6%).

**Table 1:** Baseline characteristics of included patients in different HALP score groups.

	Total	HALP-Low	HALP-High	
Variables	(n = 203)	(n = 132)	(n = 71)	p
**Age, Mean ± SD**	59.6 ± 9.7	59.4 ± 9.5	59.8 ± 10.2	0.795
**Age, n (%)**				0.773
≤ 65	137 (67.5)	90 (68.2)	47 (66.2)	
> 65	66 (32.5)	42 (31.8)	24 (33.8)	
**Gender, n (%)**				*0.011*
Female	63 (31.0)	49 (37.1)	14 (19.7)	
Male	140 (69.0)	83 (62.9)	57 (80.3)	
**Type of pathology, n (%)**				0.252
Squamous cell carcinoma	48 (23.6)	36 (27.3)	12 (16.9)	
Adenocarcinoma	139 (68.5)	86 (65.2)	53 (74.6)	
Other	16 (7.9)	10 (7.6)	6 (8.5)	
**Stage on diagnosis, n (%)**				0.058
IIIB	43 (21.2)	26 (19.7)	17 (23.9)	
IIIC	5 (2.5)	1 (0.8)	4 (5.6)	
IV	155 (76.4)	105 (79.5)	50 (70.4)	
**Pleural effusion, n (%)**				*<0.001*
No	124 (61.1)	69 (52.3)	55 (77.5)	
Yes	79 (38.9)	63 (47.7)	16 (22.5)	
**History of smoking, n (%)**				0.728
No	111 (54.7)	71 (53.8)	40 (56.3)	
Yes	92 (45.3)	61 (46.2)	31 (43.7)	
**Chemotherapy regimens, n (%)**				0.496
Pemetrexed + platinum	91 (44.8)	62 (47)	29 (40.8)	
Paclitaxel + platinum	50 (24.6)	28 (21.2)	22 (31)	
Gemcitabine + platinum	15 (7.4)	10 (7.6)	5 (7)	
Etoposide + platinum	47 (23.2)	32 (24.2)	15 (21.1)	
**Therapeutic response, n (%)**				0.95
PD	25 (12.3)	16 (12.1)	9 (12.7)	
PR	60 (29.6)	40 (30.3)	20 (28.2)	
SD	118 (58.1)	76 (57.6)	42 (59.2)	
**BMI, n (%)**				0.165
Under-weight	23 (11.3)	19 (14.4)	4 (5.6)	
Normal weight	116 (57.1)	72 (54.5)	44 (62)	
Overweight	64 (31.5)	41 (31.1)	23 (32.4)	

BMI, Body Mass Index; HALP, Hemoglobin-Albumin-lymphocyte-Platelet; PR, Partial Remission; PD, Disease Progression; SD, Stable Disease. Leaning lettering words stand for statistically significant differences.

### Association between HALP score and clinical characteristics

As shown in Table 1, there were no significant differences in age, pathological type, stage at diagnosis, history of smoking, regimen of chemotherapy, BMI, and therapeutic response among patients with different HALP scores. However, male patients usually had markedly higher HALP scores (p = 0.011), and patients with pleural effusion had significantly lower HALP scores (p < 0.001) (Table 1).

### PFS/OS of patients in different groups

**Figure 3:**
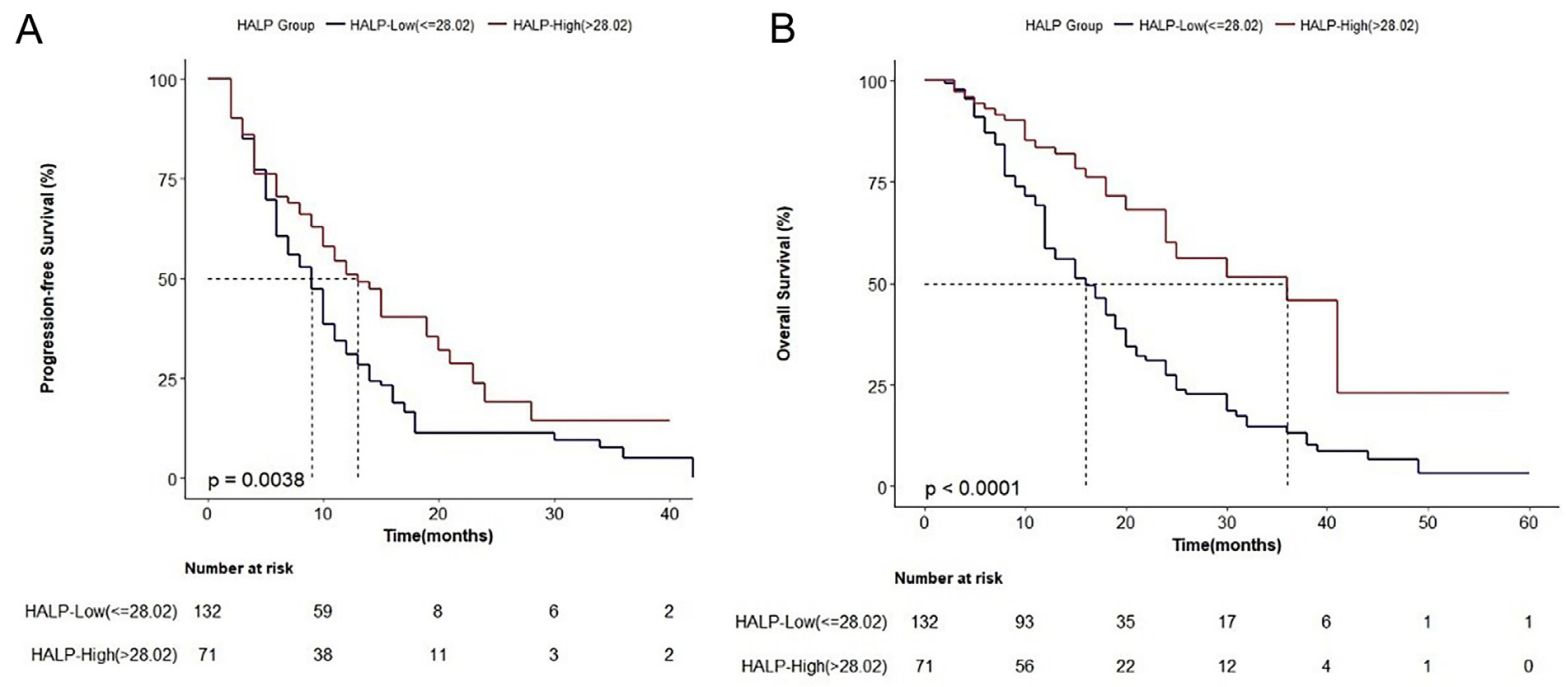
Kaplan-Meier analysis of PFS (A) and OS (B) of advanced NSCLC patients according to HALP score. HALP, Hemoglobin-Albumin-Lymphocyte-Platelet.

During the follow-up period (median: 16 months; range: 2 to 60 months), 123 (60.59%) patients died. According to Kaplan-Meier curves and log-rank test, the median PFS was 10 months, and the median OS was 19 months. Patients in the HALP-High group (13 m vs. 9 m, p = 0.0038) had significantly longer PFS. In addition, the OS in patients of the HALP-High group was more than twice that in the HALP-Low group (36 m vs. 16 m, p < 0.0001) (Fig. 3 A‒B).

### Subgroup analysis based on TNM stage and ECOG performance status

**Figure 4:**
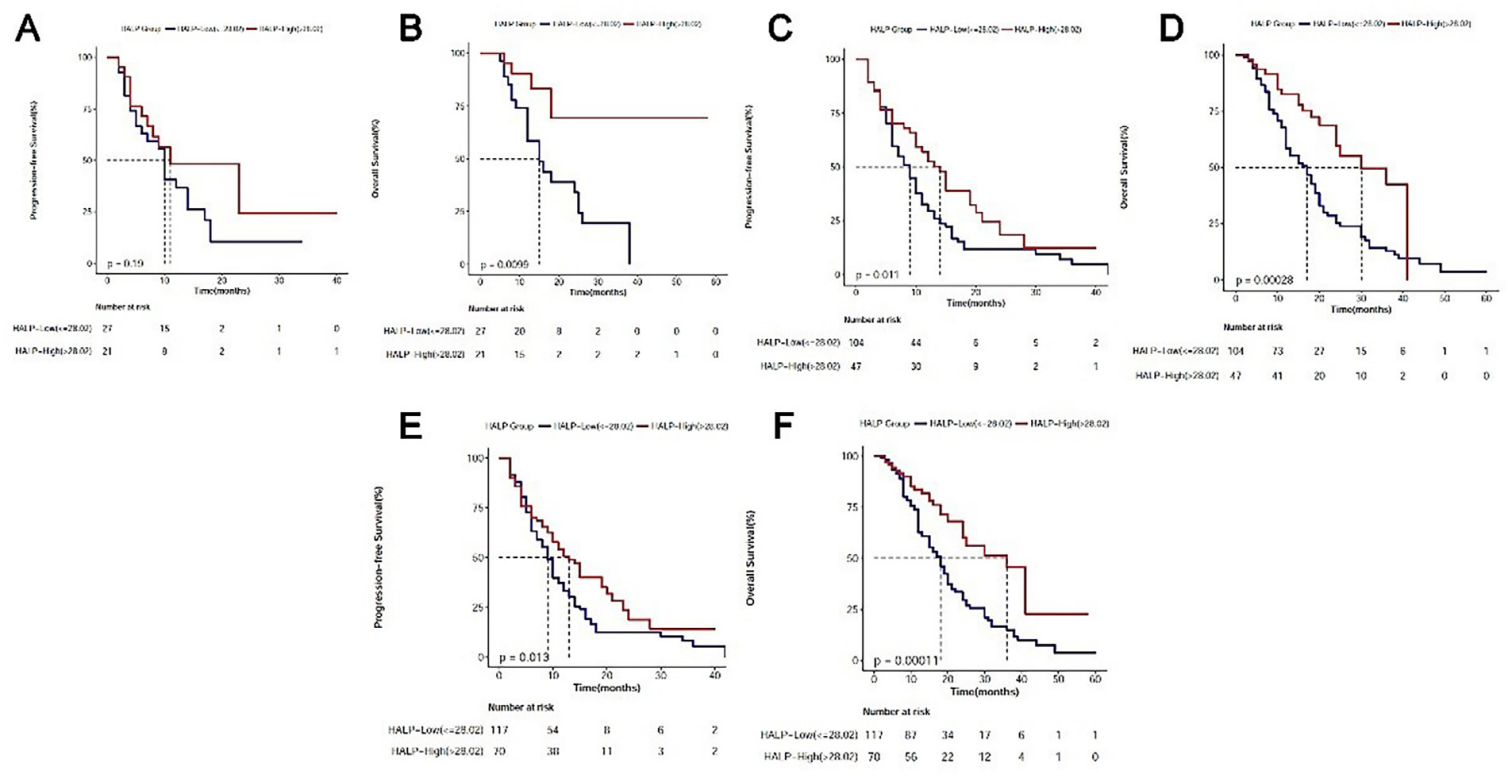
Kaplan-Meier analysis of PFS and OS of advanced NSCLC patients according to HALP score. Subgroup analysis based on the TNM stage and ECOG performance status. Stage III: PFS(A); OS (B); stage IV: PFS (C); OS (D). ECOG performance status 0‒1: PFS (E); OS (F).

Subgroup analysis of patients was further performed based on the TNM stage and Eastern Cooperative Oncology Group Scale of Performance (ECOG-PS).^21^ Results showed both stage III and IV patients in the HALP-High group had significantly longer OS (p < 0.05); stage IV patients in the HALP-High group also had markedly longer PFS (p < 0.05); no significant differences were observed in the OS and PFS in stage III patients (p = 0.16). In addition, patients with ECOG-PS of 0‒1 in the HALP-High group had significantly longer PFS and OS; there were only 16 patients with ECOG-PS of 2, and survival benefit was not observed in the PFS and OS in these patients (Fig. 4 A‒F).

### Univariate and multivariate analyses

Male gender was related to worse OS in the univariate analysis (p = 0.027), but gender has no relationship with PFS. Low HALP score, PD, ALB and LYM were all closely related to shorter PFS and OS (p < 0.001) (Table 2, Table 3).

**Table 2:** Univariate and multivariate analyses of OS.

	Univariate analysis		Multivariate analysis	
Variables	HR (95%CI)	p	HR (95%CI)	p
Gender				
Male	*1.57 (1.05*‒*2.33)*	*0.027*	*2.16 (1.32*‒*3.52)*	*0.002*
Female				
Age (years)				
> 65	1.25 (0.86‒1.82)	0.25	1.1 (0.73‒1.67)	0.649
≤ 65				
BMI (kg/m^2^)				
< 18.5	Reference			
18.5‒23.9	1.03 (0.57‒1.86)	0.928		
≥ 24	0.99 (0.53‒1.86)	0.985		
Smoking history				
Yes	1.39 (0.97‒1.98)	0.073	1.03 (0.67‒1.59)	0.876
No				
Type of pathology				
Squamous cell carcinoma	Reference			
Adenocarcinoma	0.89 (0.59‒1.36)	0.594		
Other	1.36 (0.70‒2.67)	0.364	*2.16 (1.07*‒*4.39)*	*0.032*
Stage at diagnosis				
IV	1.18 (0.75‒1.84)	0.476	1.09 (0.67‒1.77)	0.729
IIIB+IIIC				
Pleural effusion				
Yes	1.20 (0.83‒1.71)	0.33	1.12 (0.75‒1.67)	0.574
No				
Chemotherapy regimen				
Pemetrexed + platinum	Reference			
Paclitaxel + platinum	0.93 (0.60‒1.44)	0.737	1.11 (0.68‒1.81)	0.668
Gemcitabine + platinum	0.55 (0.25‒1.22)	0.144		
Etoposide + platinum	0.85 (0.54‒1.34)	0.491		
Treatment response				
PD	*2.20 (1.39‒3.47)*	*<0.001*	*2.05 (1.24‒3.4)*	*0.005*
ORR (PR+SD)				
HALP score				
> 28.02	*0.38 (0.24‒0.60)*	*<0.001*	*0.3 (0.19‒0.49)*	*<0.001*
≤ 28.02				
PLT (/L)	1.00 (1.00‒1.00)	0.249		
ALB (G/L)	*0.86 (0.83‒0.89)*	*<0.001*		
LYM (/L)	*0.51 (0.38‒0.69)*	*<0.001*		
HB (g/L)	1.01 (1.00‒1.02)	0.219		

ALB, Albumin; BMI, Body Mass Index; CI, Confidence Interval; HALP, Hemoglobin-Albumin-Lymphocyte-Platelet; HB, Hemoglobin; HR, Hazard Ratio; LYM, Lymphocytes; ORR, Objective Response Rate; OS, Overall Survival; PD, Disease Progression; PLT, Platelets; PR, Partial Remission, SD, Stable Disease. Italic: statistically significant.

**Table 3:** Univariate and multivariate analyses of PFS.

	Univariate analysis		Multivariate analysis	
Variables	HR (95% CI)	p	HR (95% CI)	p
Gender				
Male	1.23 (0.87, 1.74)	0.239	1.33 (0.88∼2.01)	0.179
Female				
Age (years)				
> 65	1.00 (0.71, 1.41)	0.994	0.82 (0.57∼1.19)	0.308
≤ 65				
BMI (kg/m^2^)				
< 18.5	Reference			
18.5‒23.9	1.00 (0.60, 1.69)	0.989		
≥ 24	0.90 (0.52, 1.56)	0.702		
History of smoking				
Yes	1.18 (0.86, 1.61)	0.309	1.03 (0.71∼1.48)	0.894
No				
Type of pathology				
Squamous cell carcinoma	Reference			
Adenocarcinoma	0.87 (0.6, 1.27)	0.478		
Other	0.98 (0.52, 1.83)	0.94	1.28 (0.66∼2.46)	0.461
Stage at diagnosis				
IV	1.17 (0.79, 1.71)	0.432	1.11 (0.72∼1.71)	0.624
IIIB+IIIC				
Pleural effusion				
Yes	1.01 (0.73, 1.39)	0.974	1.02 (0.72∼1.43)	0.929
No				
Chemotherapy regimen				
Pemetrexed + platinum	Reference			
Paclitaxel + platinum	0.89 (0.60, 1.31)	0.547	1.04 (0.69∼1.57)	0.842
Gemcitabine + platinum	0.72 (0.37, 1.39)	0.321		
Etoposide + platinum	0.78 (0.52, 1.18)	0.236		
Therapeutic response				
PD	2.54 (1.63, 3.95)	*<0.001*	*2.76 (1.69∼4.5)*	*<0.001*
ORR (PR+SD)				
HALP				
> 28.02	0.6 (0.42, 0.85)	*0.004*	*0.54 (0.37∼0.79)*	*0.001*
≤ 28.02				
PLT (/L)	1.00 (1.00, 1.00)	0.516		
ALB (G/L)	0.93 (0.9, 0.95)	*<0.001*		
LYM (/L)	0.82 (0.68, 0.99)	*0.041*		
HB (g/L)	1.00 (0.99, 1.01)	0.773		

ALB, Albumin; BMI, Body Mass Index; CI, Confidence Interval; HALP, Hemoglobin-Albumin-Lymphocyte-Platelet; HB, Hemoglobin; HR, Hazard Ratio; LYM, Lymphocytes; ORR, Objective Response Rate; PD, Disease Progression; PFS, Progression-Free Survival; PLT, Platelets; PR, Partial Remission, SD, Stable Disease. Leaning lettering words stand for statistically significant differences.

In the multivariate analysis, male gender (HR = 2.16, p = 0.002), other pathological types (HR = 2.16, p = 0.032) and PD (HR = 2.05, p = 0.005) were the independent factors associated with shorter OS. PD (HR = 2.76, p < 0.001) was closely related to shorter PFS. Meanwhile, high HALP score was an independent prognostic factor for longer PFS (HR = 0.54, p = 0.001) and OS (HR = 0.30, p < 0.001) (Tables 2 and 3).

### Prognostic nomogram for OS

**Figure 5:**
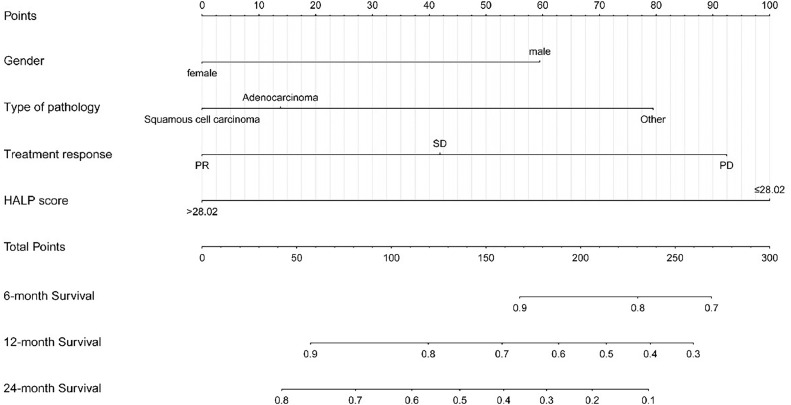
A nomogram for predicting OS. (First, using this nomogram, individualized patient data are positioned on each variable axis, then a vertical line is positioned upward to find the specific score for that indicator. Next, the specific scores for the indicator were located, and the individual scores were summed to locate the total score on the total score axis. Finally, from the localized point on the total score axis, a vertical line is plotted downward to intersect the 6-, 12-, and 24-month survival lines to determine the probability of survival at the specific time point). OS, Overall Survival.

Based on the results of multivariate regression analysis, the prognostic nomogram for OS was established based on three parameters (gender, pathological type, and outcome) and HALP score (Fig. 5). The C-index for OS prediction was 0.7036 (95% CI 0.643 to 0.7643).

### Comparison of predictive accuracy between the nomogram and HALP score alone

**Figure 6:**
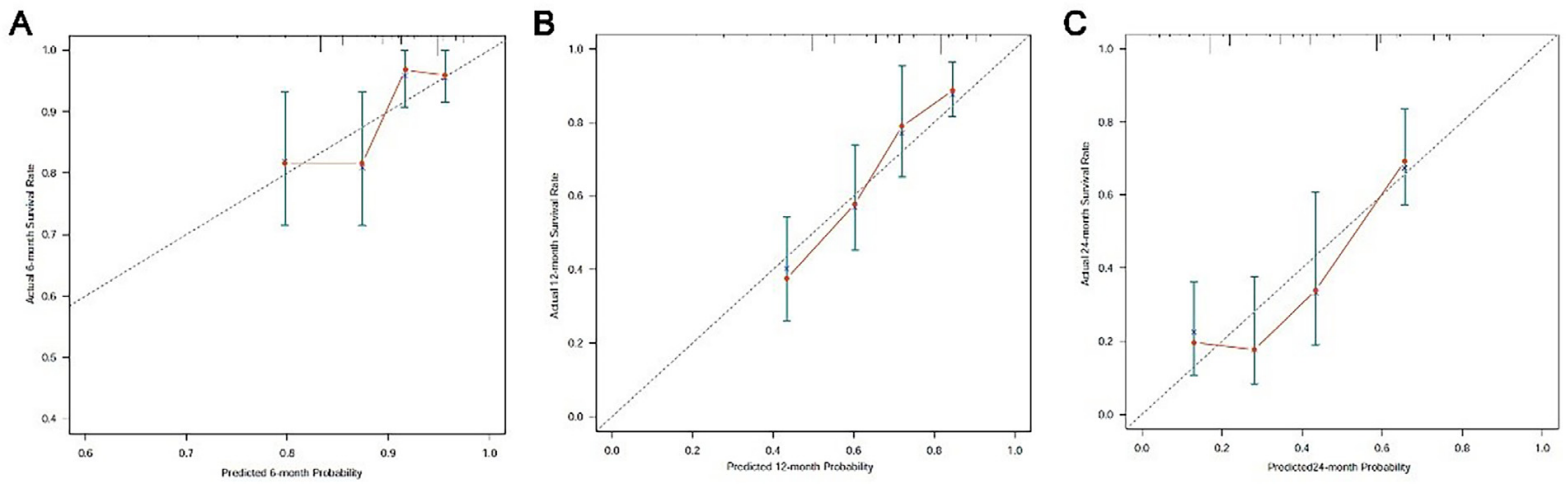
Calibration curve for predicting OS rate at 6 (A), 12 (B) and 24-months (C). OS, Overall Survival.

As shown in Figure 6, the predictive performance of the nomogram and HALP score alone for the prognosis of advanced NSCLC patients was compared. For the prediction of 12-month survival, the AUC of HALP alone was 0.612 (95% CI 0.547‒0.678) and the AUC of the nomogram was 0.782 (95% CI 0.714‒0.850). The AUC of the new prediction model was significantly larger than that of HALP alone; similar results were also observed in the prediction of 6- and 24-month outcomes.

### Validation of the nomogram

**Figure 7:**
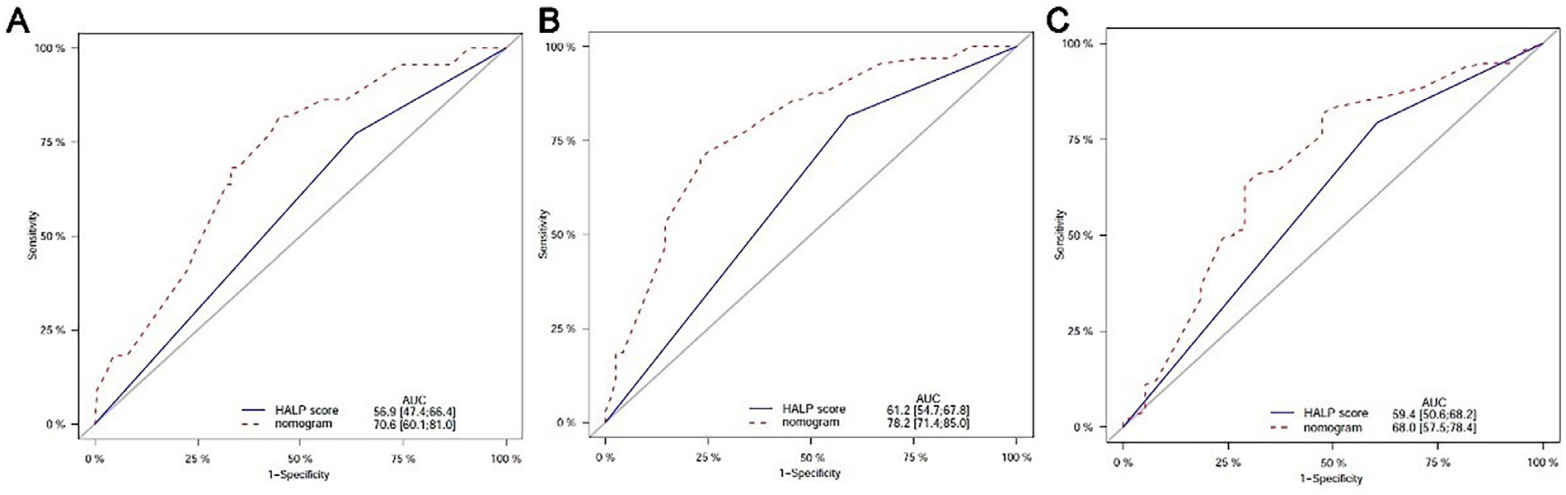
Comparison of predictive accuracy between nomogram and HALP score alone by ROC curves at 6 (A), 12 (B) and 24-months (C).

The calibration curve was performed to validate the predictive performance for OS in patients with advanced NSCLC. The calibration curve showed good agreement between predicted and actual survival at different time points. Especially, the optimal agreement in the predicted and actual survival was observed at 12 months (Fig. 7).

## Discussion

NSCLC has been an important threat to public health worldwide.^22^ With the continuous improvement of molecular detection technology, increasing pathogenic genes have been identified for lung cancer. Patients positive to pathogenic genes can benefit from the targeted therapy in the quality of life and OS.^23^ However, studies with large sample sizes have shown that 36% of advanced NSCLC patients have undetected sensitive pathogenic gene mutations.^24^^,^^25^ For these patients, platinum-based combination chemotherapy is still recommended as the first-line therapy. Nonetheless, the therapeutic response is poor in these patients, and the median survival time in these patients is still shorter than 12 months. Therefore, it is imperative to develop new predictive model with simpler and more efficient parameters for the assessment of prognosis of advanced NSCLC patients, which is helpful for the development of individualized treatment.

Inflammation is one of the hallmark features of cancers.^26^ Persistent chronic inflammation has been shown to be involved in the occurrence and development of cancers, including metastasis and therapeutic resistance. Malnutrition is another important factor related to tumor susceptibility. Numerous inflammatory and nutritional markers have previously been proposed to be correlated with lung cancer prognosis, such as Platelet-to-Lymphocyte Ratio (PLR) and Platelet-to-Albumin Ratio (PAR).^27^^,^^28^ HALP as a relatively new integrative indicator of nutritional and chronic inflammatory status was first proposed by Chen et al. in a study investigating the prognosis of gastric cancer patients.^17^

In the present retrospective study, the relationship of HALP score with PFS and OS was investigated in the advanced NSCLC patients receiving platinum-based combination therapy. Moreover, the authors for the first time established a nomogram based on the HALP score, aiming to more accurately predict the survival of these patients.

Available studies focusing on the association between HALP score and early resectable lung cancer have indicated that OS is much longer in the HALP-High group as compared to the HALP-Low group (p < 0.001).^16^ A study conducted in 362 NSCLC patients receiving adjuvant chemotherapy revealed that a lower HALP score was related to shorter Disease-Free Survival (DFS) (p < 0.01) and OS (p = 0.02).^15^ In addition, subgroup analysis showed that, for patients with locally advanced or metastatic NSCLC, a lower HALP score predicted shorter OS (p = 0.01) and DFS (p = 0.04).

These outcomes were highly in agreement with the results in the present study: shorter PFS was closely related to low HALP score (p < 0.005); patients in the HALP-High group had significantly longer OS (36 m vs. 16 m). This also confirms the strength and accuracy of the present study's predictions using the HALP score.

TNM is one of the most important factors guiding treatment and predicting prognosis. Studies have shown that T, N, and M stages are all associated with survival outcomes in lung cancer patients, and the higher the TNM stage, the worse the survival prognosis is.^29^ A retrospective study showed a consistent prognostic correlation of HALP levels with different stages in metastatic renal cell cancer patients.^30^ The subgroup analysis also yielded similar results: in stage III NSCLC patients, patients with high HALP scores had longer OS, while in stage IV patients, a high HALP score was associated with longer PFS and OS. ECOG-PS is a measure of the functional and self-care capabilities of a patient.^31^ It is important for decision-making and has been a prognostic indicator in advanced malignancy patients. ECOG-PS is commonly used to reflect physical condition and self-care ability, and the guidelines state that patients with ECOG-PS scores of 0‒2 can benefit from standard chemotherapy-containing therapy. In this study, a higher HALP score was also associated with longer PFS and OS in patients with ECOG-PS score of 0‒1. In contrast, in patients with an ECOG-PS score of 2, the relationship between the HALP score and survival outcome was not observed, which might be ascribed to the small sample size in these patients with ECOG-PS score of 2.

In addition, male patients had higher HALP scores than female ones (p = 0.011). This may be explained as that Hb level is incorporated into HALP score and male patients usually have higher mean Hb and therefore higher HALP score. Additionally, lung cancer with pleural metastases is classified as M1a and stage IV based on the TNM staging system, and patients with pleural metastases had significantly lower HALP scores in the present study (p < 0.001). Usually, tobacco dependence is often observed in some lung cancer patients. In the present study, the proportion of smokers (45.3%) was similar to that of non-smokers (54.7%) because of the small sample size or inclusion of more female patients (31%). Smoking abstinence remains one of the most effective measures to reduce the incidence or risk of lung cancer.

In univariate and multivariate analyses, other pathological types, PD, and HALP scores were independent factors related to shorter OS. Similar results were reported in a recent study in which male gender and lower HALP scores were also related to shorter OS and DFS in patients undergoing postoperative adjuvant chemotherapy for NSCLC.^15^ Furthermore, a new nomogram was established based on the HALP score in the present study and was used to successfully predict the survival outcome of advanced NSCLC patients at 6-, 12-, and 24-months. According to the calibration curves, the 12-month overall survival prediction line best matched the reference line. In addition, the comparison of ROC and C-index between the two models showed that the predictive ability of this new nomogram model was superior to that of the HALP score alone.

The present study for the first time found that the lower HALP score predicted a poor prognosis in advanced NSCLC patients receiving platinum-based chemotherapy. Nevertheless, there were several limitations in this study. First, this was a retrospective study, and the selection and information biases can't be excluded in the establishment of new nomogram with data from a single center. In this regard, patients with pathogenic mutations or concomitant use of immune and anti-angiogenic drugs were excluded from this study. Second, the sample size was still small, and patients with complete efficacy evaluation and laboratory examination data were included in this study. In addition, the survival status of these patients was followed up by telephone, WeChat, and a medical record system. Thus, more multicenter prospective studies with large sample sizes are warranted to confirm these findings in the future.

## Conclusions

The present study indicates that the HALP score may be an accurate and readily available prognostic factor for PFS and OS in advanced NSCLC patients receiving standard first-line platinum-based regimens. A higher HALP score (> 28.02) predicts better PFS and OS. Furthermore, a new nomogram is established based on the HALP score and its predictive performance for OS is better than that of the HALP score alone. This new nomogram may assist clinicians in the initial prediction of survival in these patients.

## Conflict of interest

The authors declare no conflicts of interest.
